# Summary of Discussions From the 2019 OECD Conference on RNAi Based Pesticides

**DOI:** 10.3389/fpls.2020.00740

**Published:** 2020-05-29

**Authors:** Michael L. Mendelsohn, Achim Gathmann, Dimitra Kardassi, Magdalini Sachana, Emily M. Hopwood, Antje Dietz-Pfeilstetter, Stephani Michelsen-Correa, Stephen J. Fletcher, András Székács

**Affiliations:** ^1^Biopesticides and Pollution Prevention Division, Office of Pesticide Programs, United States Environmental Protection Agency, Washington, DC, United States; ^2^Federal Office of Consumer Protection and Food Safety, Department of Plant Protection Products, Unit Environment, Braunschweig, Germany; ^3^Pesticide Peer Review Unit, Scientific Evaluation of Regulated Products Directorate, European Food Safety Agency, Parma, Italy; ^4^Environment Health and Safety Division, Environment Directorate, Organisation for Economic Cooperation and Development, Paris, France; ^5^Health Evaluation Directorate, Pest Management Regulatory Agency, Health Canada, Ottawa, ON, Canada; ^6^Institute for Biosafety in Plant Biotechnology, Julius Kühn-Institut, Federal Research Centre for Cultivated Plants, Braunschweig, Germany; ^7^Science and Technology Policy Fellow, American Association for the Advancement of Science, Washington, DC, United States; ^8^Centre for Horticultural Science, Queensland Alliance for Agriculture and Food Innovation, University of Queensland, St. Lucia, QLD, Australia; ^9^Agro-Environmental Research Institute, National Agricultural Research and Innovation Centre, Budapest, Hungary

**Keywords:** double stranded RNA, RNA interference, pest control, regulation, gene silencing, environmental risk assessment, non-target organisms

## Abstract

RNA interference (RNAi) is a biological process in which double-stranded ribonucleic acid (dsRNA) molecules inhibit protein expression. In recent years, the application of dsRNA has been used in the development of agricultural products for pest control. The 2019 Organisation for Economic Cooperation and Development (OECD) Conference on RNAi Based Pesticides (“the Conference”) brought together academic, industry, and government experts in various aspects of RNAi to discuss the current state of knowledge and topics to help in developing considerations for risk assessment. The Conference focused on environment, with some discussion of human health. Along with presentations on the use of dsRNA-based products in agriculture, government regulation, risk assessment, and a background on the Draft OECD Working Paper on “Considerations for the Environmental Risk Assessment of the Application of Sprayed or Externally Applied dsRNA-Based Pesticides” (“OECD Working Paper”), the Conference included panel discussions from presenters at the end of each session and a larger discussion session with Conference participants on the environmental fate of dsRNA, non-target organism (NTO) risk assessment, and human health risk assessment. This paper summarizes input from presenters and Conference participants during these discussions. Key considerations from these discussions have already been incorporated into the OECD Working Paper, that once finalized and published, will facilitate regulators in evaluating externally applied dsRNA-based products for potential environmental risks.

## Background

RNA interference (RNAi) is a biological process in which double-stranded ribonucleic acid (dsRNA) molecules inhibit protein expression, typically by triggering the enzymatic cleavage of specific messenger RNA (mRNA) molecules which are templates for protein synthesis (Fire et al., 1998; Agrawal et al., 2003). In this process, short interfering RNAs (siRNAs) derived from Dicer-mediated cleavage of long dsRNA initiate destruction of an mRNA through complementary base-pairing (Elbashir et al., 2001). This sequence-specific mode of action has been harnessed for the development of genetically modified (GM) corn for corn rootworm control (Bolognesi et al., 2012) and for targeted human and animal therapies (Vaishnaw et al., 2010). Pest control may be achieved by inducing RNAi through topical application (e.g., spraying) of dsRNA (with a nucleotide sequence developed to target a specific gene from pest or pathogen species) onto plants (San Miguel and Scott, 2015; Joga et al., 2016; Cai et al., 2018; Zotti et al., 2018). To date, no topically applied dsRNA-based pesticides have been approved for use. Guidance on regulating this technology is needed since the commercialization of these products would have implications for existing policies.

As a part of its general target of improving global economic performance, the OECD aims to establish evidence-based international standards and to find solutions to a range of social, economic and environmental challenges. OECD’s Co-operative Research Programme (CRP), formed by 24 countries of the 36 OECD member nations, sponsors fellowships and conferences on biological resource management for sustainable agriculture to strengthen scientific excellence and to inform future policy decisions related in the areas of agriculture, food, fisheries and forests. To address the feasibility of siRNAs as external plant protection agents, OECD CRP organized a 2.5-day conference (held on 10–12 April 2019 in Paris, France), and summarized the current state of knowledge and ongoing developments that are relevant for the regulation of dsRNA-based pesticides. There were 57 participants from academia (∼22%), industry (∼21%), and government (∼57%). Invited speakers included experts in various aspects of RNAi, and their presentations summarized product developments, environmental fate, exposure to externally applied dsRNA in NTOs, lessons from human therapeutic use of dsRNA, and key points from previous regulatory reviews of dsRNA-based crop traits.

## Discussion

The summaries captured here represent the varied input from multiple participants during the Conference discussion sessions and do not necessarily reflect consensus views. In the interest of space, comments from participants have been consolidated and edited for length and clarity. Content in the text is attributed using the sector of the speaker affiliation in parentheses.

### Session 1: Summary of the State of the Art: dsRNA Product Use in Agriculture

A summary of the scientific background of dsRNA products in agriculture was provided by scientists from academia, industry, and government.

The use of bioinformatics analyses for predicting off-target effects and unintended gene silencing in NTOs was the primary focus of the group discussion. The participants noted that sequence homology between a dsRNA and a mammalian transcript does not necessarily lead to a silencing effect. In mammals, there is a high rate of potential matches of a dsRNA with a target gene for any dsRNA of ∼300 base pairs (bp) or more. This is generally due to the low target site specificity in mammals, where a short (7–8 bp) ‘seed’ region of an siRNA is sufficient for binding to transcripts and subsequent silencing. The disconnect between predicted dsRNA/transcript sequence homology and physiological effects in part is due to barriers that prevent dsRNA uptake by mammalian cells and the relatively low abundance of discrete siRNAs derived from a single dsRNA. The participants acknowledged that multiple mechanisms underlie the reported disconnect and noted differences between organisms that share a common microRNA/siRNA pathway (i.e., mammals) and those with different pathways (i.e., insects).

The concentration of individual siRNAs is another important parameter for predicting off-target effects. The risk to NTOs may be minimal if individual siRNAs are below the femtomolar range. Off-target effects may be limited by the use of low concentrations of a dsRNA preparation yielding correspondingly low concentrations of each unique individual siRNA, in contrast to a large amount of a single siRNA. Another advantage of longer dsRNAs (>60 bp) is the ability to design a molecule which can target several transcripts at a time.

A comparison of plant-produced dsRNA to spray applications of dsRNA for pest control was discussed. Generally, the dsRNA produced in genetically modified plants (GMPs) is processed within the plant into siRNAs, which are subsequently 3′ end-methylated and therefore more stable. The GMP strategy could result in less contact with NTOs and less potential for drift, runoff, or movement of the dsRNA to other environmental compartments compared to spray applications. In contrast, the potential for environmental spread via transgene escape to non-GM counterparts is not evident for spray applied dsRNAs. However, multiple factors (e.g., specificity, route of exposure, responsiveness to environmental RNA) were considered more relevant for risk assessment than a very general comparison of the method of deployment of RNAi in agriculture.

In this context, the question of when to quantify the dsRNA for exposure measurement arose, especially given that dsRNA-based pesticides may take longer to display efficacy than conventional pesticides. The group stressed that a time profile for dsRNA exposure is needed. For spray applications, there was consensus that formulation is an important factor to be addressed in the OECD Working Paper because it is essential to the stability and uptake of dsRNA.

### Session 2: Summary of Regulatory and Risk Assessment Experience With dsRNA-Based Products

While research on the mechanisms of action of dsRNA is important, the group consensus was that the importance for risk assessment lies in identifying potential hazards rather than the mechanisms underlying those hazards. Additional emphasis should be placed on the selection of NTOs to conduct toxicity testing for ecological risk assessments, paying close attention to the possibilities for environmental and systemic RNAi in test species to avoid false negative results.

The role of bioinformatics in assessing potential hazards was a recurring theme. The use of bioinformatics is believed to have limited value in assessing off-target effects due to the variability in environmental exposure across organisms, barriers to systemic exposure, and differences in RNAi machinery between organisms. There was group consensus that sequence information alone should not and cannot be used as the sole predictor of effects on NTOs. However, while bioinformatics is of some limited value for hazard evaluation, it is critical in the design phase of dsRNA products and for selecting NTOs to study.

Protocols for addressing hazards with dsRNA-based products require revision compared to those for conventional pesticides because dsRNA-based products may take longer to display efficacy. Any evaluation needs to account for this time lag by extending the study period. Additionally, any evaluation of a dsRNA-based pesticide should address the degradation of the dsRNA over time. Effective use of a dsRNA-based pesticide may require a recharging application as degradation is known to occur.

Issues of public perception of dsRNA were raised. dsRNA-based pesticides are molecular biological products, but not considered as genetic modification technology since the nucleotide sequences in dsRNA-based pesticides do not code for protein and are not inserted into the genome and are not heritable like transgenes (Academia). (Authors note. Therefore, they do not fall under the scope of various gene technology regulations in certain OECD countries regardless of their slightly different definitions of GM organisms.) However, there is concern that the public may misunderstand RNAi-based pesticides as a “new genomic technique” and this may create an obstacle to public acceptance (Academia). Participants discussed this topic from a variety of perspectives (Table 1, Question 1).

**TABLE 1:** Responsive discussions to questions raised by participants.

*1. How can governments, regulators, and industry convey ideas about this new technology to the public? What is the best way to market RNAi technology so that it is not incorrectly perceived as genetic modification? How can this clear distinction be communicated openly?*	
•	Researchers, regulators, and industry need to be transparent and accessible in communications, especially when mentioning “DNA”, “RNA”, or “genes” (Academia).
•	There was consensus about the need to initiate conversations with the public early. Academic scientists highlighted the need to engage in dialogue with social policy, regulatory agencies, and social scientists.
•	Participants discussed this issue at some length, recognising that, regardless of scientific consensus on safety, balancing the need for transparency with communicating scientific uncertainty and risk to the public is a challenge.
•	In Europe, the definition of genetic modification specifies living organisms. If dsRNA is purified, it cannot be legally defined as genetically modified (Government Regulator). However, the public may not see this distinction (Government Regulator).
•	An industry scientist pointed out that part of the issue with public acceptance of genetically modified plants is that the field is perceived to be dominated by large companies (Industry). This may be less of an issue with RNAi-based products because of the cost for producing this technology is substantially lower and broad interest from diverse developers exists (Industry).
*2. Are there safety concerns with nanoclay particles?*	
•	A range of nanomaterials and their safety profiles have been investigated (Academia). An example of a nanoclay is layered double hydroxide (LDH), which has characterized nanometrology properties and is biocompatible and biodegradable (Academia).
•	Plant cell walls have size exclusion limits. Because the LDH component of BioClay (a complex of LDH and dsRNA) ranges from 20 – 80 nm, which exceeds the pore size on plant cell walls (< 5 nm), it is likely excluded from entering plant cells and may just act as a carrier that slowly releases dsRNA on the plant surface (Academia).
*3. Is there evidence of dsRNA entering a plant’s RNAi system or down-regulating an endogenous gene?*	
•	A number of papers report dsRNA-generated systemic resistance to viruses and fungi without mechanical inoculation at the point of dsRNA application, implying that dsRNA can enter the plant through an intact surface, possibly via the stomata. The nature of this process is undetermined.
•	As topically-applied dsRNA has repeatedly generated successful systemic virus resistance, this suggests that dsRNA enters the plant and is taken up by the RNAi system.
•	Participants were not aware of any publications that demonstrate dsRNA spray-induced reduction of endogenous gene expression. The mechanism for long distance transport of topical dsRNA-derived silencing signals is undetermined.

### Session 3: Discussion Themes

#### Environmental Fate

The potential for formulation to increase persistence of a dsRNA product requires special consideration regarding its environmental fate. The European Union (EU) has protocols to address products that persist, and these protocols could be relied on if a formulation increases dsRNA persistence. The need to reapply a dsRNA product with low environmental persistence may increase exposure to organisms that typically require repeated contact with a pesticide.

The group discussed the need to clarify whether the active ingredient or the formulation is tested both in pre- and post-market assessments. A focus on biologically based analytical methods rather than studies designed solely for understanding the chemistry was proposed.

In general, environmental fate and exposure considerations for various types of use patterns (e.g., foliar sprays, seed treatment) are the same for dsRNA as for conventional pesticides. Industry members suggested that a product or active ingredient should not be assessed differently than another similar product just because they have a different formulation (Industry). Additional industry input suggested that the formulated product should always be used in field trials. Regulators from the United States Environmental Protection Agency (US EPA) and the EU both expressed concern about exposure routes and how testing requirements may change with different formulations (Table 2). Application of a hazard paradigm for protected species and the ecosystem as well as the use of the endpoints already established for the risk assessment of conventional pesticides was discussed.

**TABLE 2:** Comparison of comments on testing for environmental assessments from various governmental or multi-governmental bodies.

European Union (Two step approach)		United States Environmental Protection Agency
First step: The European Food Safety Authority (EFSA) assesses an active substance including one representative formulation that the applicant proposes. The active substance will be authorized by the EU Commission.	Second step: Member State assesses each final plant protection product whose active ingredient has been authorized in the first step. The basic consideration is that differences between product formulations can alter its environmental behavior and ecotoxicological potential.	The US EPA uses a case-by-case approach and a tiered risk assessment similar to their method for evaluating biochemical pesticides.

Concern about how the RNA sequence may change degradation rates was raised (Industry). However, in the case of a naked dsRNA, the degradation kinetics should be similar regardless of the sequence (Industry). Industry members were asked what protocols they plan to use to assess persistence. Bayer plans to look at degradation in the formulated versions of their products (Industry). The use of established laboratory protocols for studying the persistence of *Bacillus thuringiensis* (Bt) proteins was proposed (Industry).

Both industry and academic participants emphasized that a relatively large body of literature exists about the mechanism of action for dsRNA relative to conventional pesticides. Concerns about the impact of additional data requirements on creating a regulatory burden for industry were raised by industry and academic partners (Industry; Academia). A discussion ensued on the potential for extrapolating environmental data obtained with one dsRNA product to others, and how much information is needed to reach a satisfactory understanding of environmental fate. A definitive conclusion was not reached.

#### Non-Target Organisms (NTOs)

The potential for exposure and responsiveness to environmental RNAi were seen as the first parameters to consider in the risk assessment of external dsRNA applications before looking at sequence data.

The potential uptake of dsRNA by mammals after oral exposure is likely to be low due to substantial barriers in the oral and dermal uptake pathways. Research on barriers to invertebrate oral and topical uptake is limited. Barriers to uptake identified in mammals are assumed to apply across vertebrate species because the molecular mechanisms show conservation from fish to mammals (Academia); similarly, the literature shows a high degree of conservation in the vertebrate digestive system (Industry). Two regulatory studies exposed mice and rats to high doses of dsRNA and no effects were observed (Industry). However, pharmaceutical data, often derived from studies using mice, are based on peritoneal administrations (i.e., injection) that are less relevant to the exposure pathways of dsRNA-based pesticides (i.e., ingestion and inhalation) (Industry). For insects, a point was raised about the lack of “negative data” in the published literature (i.e., when exposure to dsRNA did not lead to a biologically meaningful effect) (Academia). While it may be possible to generalize the applicability of barriers to dsRNA uptake identified in mammals to other vertebrates, it is currently not possible to predict responsiveness across invertebrate taxa to environmental dsRNA. The group stressed that siRNAs are not taken up by most insects and their presence in the environment generally has no biological effect (author note – short dsRNAs < 30 base-pairs have, however, been effective in some feeding assays.). However, it was noted that arthropods display variable responsiveness to long dsRNA and it is difficult to generalize susceptibility to RNAi, even among closely related arthropod species.

Before doing functional testing for investigating potential agricultural uses of dsRNA, the cellular components of RNAi in the pest or NTO of interest should be examined. Evolutionary development appears to be monophyletic for silencing and amplification of the RNA signal, and Dicer enzymes among vertebrates are fully conserved.

Bioinformatics methods are likely to be useful in the design phase of species-specific dsRNA molecules and in the identification of NTOs for testing (Industry). Several group members expressed the value of examining off-target genome and transcriptome data for regions of homology to the dsRNA, although full genomes of many target species and NTOs have not been sequenced. For instance, evaluation of *DvSnf7* dsRNA-expressing corn used two approaches to bioinformatics but was limited by the number of species that could be tested (Academia).

The length of nucleotides needed in bioinformatics alignments was discussed and a minimum contiguous match between a dsRNA and an off-target transcript of ∼19 nt was proposed, based on the scientific literature (Academia). If there are no contiguous nucleotide matches of ≥19 nt to an off-target sequence, it can be assumed that there will be no sequence-dependent effect of the dsRNA on the NTO (Academia). For a highly specific construct, it was proposed that closely related species should be tested (Academia). If a closely related species is not impacted, it is less likely a more distant one would be. The discussion on bioinformatics largely reiterates that which will be contained within the OECD Working Paper.

A challenge with using bioinformatics data is choosing the NTO of interest (Academia). Considering the regulatory process, ecological data should be used to determine which species actually live in the environment that contains the target pest (Academia). Additionally, testing organisms at various life stages may be necessary due to differential sensitivities to RNAi depending on the life stage (Government Regulator). Testing organisms from the environment of interest may not be necessary because test guidelines already specify the use of surrogate species; furthermore, testing animals from the actual environment of interest could raise concerns about biodiversity loss (Industry).

#### Human Health Risk Assessment (HHRA)

Concerns about the potential exposure of sensitive populations (i.e., children, the elderly, and the immune-compromised) to dsRNA-based pesticides were explored. No significant issues were raised regarding the safety of the technology to the health of sensitive populations. An important consideration discussed was the potential exposure of applicators and bystanders to dsRNA-based pesticides. It was noted that published studies relevant to the potential exposure of applicators and bystanders used doses of dsRNA that are substantially higher than expected exposure levels after field application in agriculture (expected to be approximately 0.5–1.0 gram dsRNA per hectare). Additionally, long dsRNAs are considered to have less potential for sequence-dependent off-target effects than siRNA. The impact of RNAi-based pesticide formulations on exposure was also discussed.

Unlike siRNA, long dsRNA has the potential to provoke an interferon response, which can be independent of the sequence. Because of this, bioinformatics is unlikely to be a useful method for informing the HHRA. It was noted that, because of the potential for an immune response, clinical studies so far have focused on shorter RNAs designed to bypass this response. Clinical data using long dsRNA may be lacking. Sequence-independent immune responses, if they occurred at all, were speculated to produce symptoms similar to mild influenza.

Evidence from human clinical studies suggests that systemic exposure of mammals to dsRNA when dsRNA is applied in the field as a pesticide may be quite low. Unfortunately, research yielding no adverse effects is not commonly published; the few available published animal studies on dsRNA include an acute and subchronic toxicity study and three repeat-dose toxicity studies. None of the studies demonstrated any adverse effects (including for sensitization and irritation) when dsRNA was administered orally. Additionally, any adverse impacts that have been observed from dsRNA were temporary and did not permanently impact the immune system.

The use of formulations to enhance cellular uptake may call for additional considerations in the HHRA, since it is likely that product formulations will increase the persistence or systemic uptake of a dsRNA active ingredient. For example, when nanomaterials are present in formulations for the purpose of enhancing cellular uptake of dsRNA, increased dermal and/or inhalation toxicity may occur. However, not all nanomaterials enhance the cellular uptake of dsRNA (Table 1, Question 2). A case-by-case consideration of the intended product’s formulation will help determine whether additional data are required.

Exogenously applied dsRNA pesticides are likely to be applied using the same methods as “conventional” pesticides. Consequently, the same exposure routes are probable. Populations most likely to be exposed are farm workers applying the product and bystanders in the target area during or following product application. The primary exposure pathways for workers and bystanders are dermal contact and/or inhalation. In the absence of a regulatory policy relating to personal protective equipment for dsRNA-based pesticides, requirements will be determined on a case-by-case basis.

For the general population, dietary consumption of treated plants and/or derived commodities will likely be the dominant exposure pathway. Regarding potential human health impacts, it is important to note that dsRNA applied to plants for pest control is not integrated into the plant genome or amplified. Rather, depending on the delivery method and on the targeted pest or pathogen dsRNA may be taken up by the plant cell and processed. Unprocessed dsRNA as well as processed siRNAs are delivered to targeted pests or pathogens to trigger the RNAi pathway within the target pest. The fate of any remaining dsRNA and whether it accumulates in plants is currently unknown (Table 1, Question 3). Even if dsRNA residue persists in the plant, there are significant physiological and biochemical barriers limiting systemic exposure to dsRNA following oral ingestion. Studies from human health research suggest that some conjugation, encapsulation or chemical modification of dsRNA is necessary to facilitate its trans-membrane movement and to reduce its otherwise rapid renal clearance. However, the potential accumulation of dsRNA in plants may warrant consideration for dietary intake following multiple applications particularly close to harvest, or when persistent formulations are applied.

An additional Working Paper addressing HHRA of dsRNA was proposed to address the development of dsRNA-based human therapeutics and the potential for human health risks based on the outcomes of these studies.

## Conclusion

The considerations arising from the Conference discussions were varied and represented multiple perspectives. The potential for exposure of NTOs as well as responsiveness to environmental RNAi were seen as the first parameters to consider in the assessment of external dsRNA applications. While sequence information is useful in the design phase and in the selection of NTOs for testing, bioinformatics cannot be used as a stand-alone predictor of off-target effects. Protocols for addressing hazard with dsRNA-based products require some revisions compared to how they are carried out for conventional pesticides (e.g., by extending the study period) since dsRNA-based products may take longer to display efficacy. Evaluations of dsRNA-based pesticides should include monitoring for degradation over time. The impact of product formulation on environmental persistence of dsRNA and uptake by non-target organisms requires consideration. While it may be possible to generalize the applicability of barriers to dsRNA uptake identified in mammals to other vertebrates, it is currently not possible to predict responsiveness across invertebrate taxa to environmental dsRNA. Considerations arising from the Conference will be incorporated into the OECD Working Paper(s) as appropriate.

## Author Contributions

EH, AD-P, SM-C, and SF were rapporteurs at the Conference. MS, MM, and SM-C consolidated the proceedings. All authors critically revised the manuscript and contributed to the writing. All authors contributed to manuscript revision, read and approved the submitted version.

## Disclaimer

The opinions expressed in this paper are the sole responsibility of the authors and do not necessarily reflect those of the OECD or of the governments of its Member countries.

## Conflict of Interest

The handling Editor is currently organizing a Research Topic with the authors MM and AS. The remaining authors declare that the research was conducted in the absence of any commercial or financial relationships that could be construed as a potential conflict of interest.
